# Retinal Microvascular Density Was Associated With the Clinical Progression of Parkinson’s Disease

**DOI:** 10.3389/fnagi.2022.818597

**Published:** 2022-02-17

**Authors:** Bei Xu, Xin Wang, Jifeng Guo, Huizhuo Xu, Beisha Tang, Bin Jiao, Lu Shen

**Affiliations:** ^1^Eye Center of Xiangya Hospital, Central South University, Changsha, China; ^2^Hunan Key Laboratory of Ophthalmology, Changsha, China; ^3^Department of Neurology, Xiangya Hospital, Central South University, Changsha, China; ^4^National Clinical Research Center for Geriatric Disorders, Xiangya Hospital, Central South University, Changsha, China; ^5^Engineering Research Center of Hunan Province in Cognitive Impairment Disorders, Central South University, Changsha, China; ^6^Hunan International Scientific and Technological Cooperation Base of Neurodegenerative and Neurogenetic Diseases, Changsha, China; ^7^Key Laboratory of Hunan Province in Neurodegenerative Disorders, Central South University, Changsha, China

**Keywords:** diagnosis, Hoehn-Yahr stage, optical coherence tomography angiography (OCTA), Parkinson’s disease, vessel density

## Abstract

**Background:**

Retinal microvascular density has been studied in neurodegenerative diseases, whereas patients with Parkinson’s disease (PD) at different clinical stages have been rarely investigated. The present study aimed to evaluate the microvascular variations in superficial retinal capillary plexus (SCP) in patients with PD on different Hoehn-Yahr (H-Y) stages by optical coherence tomography angiography (OCTA), as well as determine their relationships with clinical parameters.

**Methods:**

In total, 115 patients with PD and 67 healthy controls (HCs) were recruited. The PD group was divided into three groups based on the H-Y stage. The OCTA examination was performed in all participants, and the macular vessel density (m-VD), peripapillary vessel density (p-VD), and foveal avascular zone (FAZ) area were measured.

**Results:**

The m-VD in all regions, p-VD in center [6.1 (4.8, 6.95) mm^–1^ in healthy eyes vs. 5.1 (3.7, 6.4) mm^–1^ in patients], nasal inner (NI) [18.5 (17.8, 19.3) mm^–1^ in healthy eyes vs. 17.9 (17.1, 18.7) mm^–1^ in patients], temporal outer (TO) [19.6 (18.9, 20.2) mm^–1^ in healthy eyes vs. 19.3 (18.5, 19.7) mm^–1^ in patients] regions and FAZ area [0.36 (0.32, 0.39) mm^2^ in healthy eyes vs. 0.29 (0.26, 0.33) mm^2^ in patients] noticeably decreased in PD groups compared with HC (*p* < 0.05). Moreover, the FAZ area was suggested to decline significantly in patients with PD with H-Y I stage (*p* < 0.05), while it was more serious in the H-Y III stage in patients. Furthermore, we found that m-VD exhibited a significant negative correlation with age, disease duration, UPDRS scores, NMSS scores, and H-Y stage.

**Conclusion:**

OCTA has the potential to non-invasively detect the microvascular changes in patients with PD with different clinical stages *in vivo*, and it may be a valuable tool to monitor the PD progression.

## Introduction

Parkinson’s disease (PD) refers to the second most common disease among central neurodegenerative conditions, affecting numerous parts of the brain and showing relationships to motor dysfunction and cognitive impairment in the elderly (Kwapong et al., 2018). This disease is caused by the depletion of dopaminergic neurons. Dopamine (DA) acts as a major neurotransmitter in the retina, which critically regulates the functions of other neurochemical systems (e.g., gamma-aminobutyric acid, glycine, and glutamate in the retina) (Sengupta et al., 2018; Yıldız et al., 2019). PD is recognized as a multisystemic pathology with both motor and non-motor manifestations (de Rijk et al., 1995; de Lau and Breteler, 2006), and patients with PD develop specific visual symptoms (e.g., decreased visual acuity, deteriorated contrast sensitivity, and difficulties in visuospatial orientation) (Archibald et al., 2011; Bodis-Wollner et al., 2014; Mohana Devi et al., 2020).

The eye is regarded as a gateway to the brain, where precise functional/structural relationships are developed. Moreover, PD research primarily aims to develop methods of achieving accurate early diagnosis and to monitor disease progression in individual subjects. For this reason, if the exact cellular function/structure relationship is developed in the retina, the eye can act as a tool to conduct the pathobiological study of PD (MacCormick et al., 2015). The optical coherence tomography angiography (OCTA) enables the capture of direct non-invasive retinal imaging and the study of the microvasculature in the retina qualitatively and quantitatively. Microvascular measurements in OCTA are summarized as vessel density in the respective subfield, which facilitates the detailed study of neuronal and microvascular in PD with high-quality images (Uchida et al., 2018). Meanwhile, only a few studies reported the relationship between retinal microvascular density and PD (Kwapong et al., 2018; Shi et al., 2020). However, no consensus has been reached on retinal vascular variation in patients with PD grouped by Hoehn-Yahr (H-Y) stage with OCTA. It is hypothesized that the microvascular changes in the retina may represent differences between the diverse clinical stages, especially when the patients with PD progress from minimal to severe stage. Accordingly, this study is the first to evaluate the diagnostic significance of macular and peripapillary vessel density (m-VD and p-VD) and FAZ through OCTA in patients with PD at different clinical stages with Hoehn-Yahr staging scores. Moreover, the relationships between quantitative OCTA measures and PD clinical parameters were analyzed.

## Materials and Methods

### Enrollment of Participants

In total, 115 patients with PD from Xiangya Hospital between October and December 2019 were recruited. 67 age- and gender-matched HC were recruited from the family members of patients with PD or colleagues in the hospital of the identical period. To avoid the correlation of intrasubject inter-eyes, we selected the random eye from each subject. Prior to the participation here, all participants provided written informed consent forms. The diagnosis of PD was diagnosed in accordance with the “Movement Disorder Society (MDS) clinical diagnostic criteria for Parkinson’s disease” (Postuma et al., 2015). The respective patient was assessed at the H-Y staging scale (Goetz et al., 2004) and Unified Parkinson’s Disease Rating Scale (UPDRS; Goetz et al., 2008). Mini-Mental State Examination (MMSE) and Non-motor Symptoms Scale (NMSS) were adopted to assess non-motor symptoms. By complying with the H-Y stage, the patients with PD in this study fell to the following three groups, i.e., the H-Y I stage group, the H-Y II stage group, and the H-Y III stage group.

Demographic and neurologic data consisted of age, sex, and disease duration. All participants were between 50 and 80 years old. The exclusion criteria were presented as the patients with (1) glaucoma, age-related macular degeneration, macular hole, epiretinal membrane, refractive error [> ±3 diopters (D) sphere and ±2D cylinder], macular drusen, as well as pathological myopia, (2) diabetes and uncontrolled hypertension, (3) concomitant neurological disorders, (4) severe cardiac, pulmonary, hepatic, renal diseases, or any types of tumors, and (5) insufficient clinical data. This study was approved by the Research Ethics Committee of Xiangya Hospital, Central South University. The study abided by the Declaration of Helsinki, which was performed by complying with the approved guidelines and regulations.

### Optical Coherence Tomography Angiography Imaging

**FIGURE 1:**
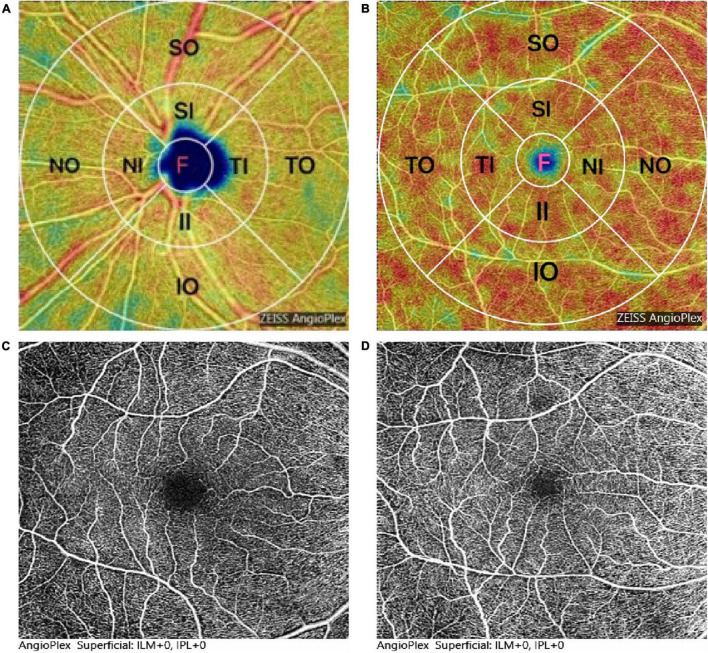
Representation of optical coherence tomography angiography (OCTA) image and measurement of the density analysis of the superficial retinal capillary plexus (SCP). **(A,B)** Map of macular and peripapillary, 1 mm central circle, representing the center area, and inner and outer rings of diameters 3 and 6 mm, respectively. The inner and outer rings were divided into four quadrants: superior, nasal, inferior, and temporal. **(C,D)** Foveal avascular zone (FAZ) area was significantly decreased in patients with Parkinson’s disease (PD) **(D)** compared to healthy controls (HC) **(C)**.

All participants underwent retina microvasculature imaging based on the Cirrus 5000 HD-OCT (Carl Zeiss Meditec, Inc., Dublin, CA, United States). The m-VD and p-VD in superficial retinal capillary plexus (SCP) was analyzed throughout the examined area of 6 mm × 6 mm that was centered on the macula and peripapillary, and they consisted of vessels from the layer of the inner limiting membrane (ILM) to the inner plexiform layer (IPL). In addition, the vessel density was measured in nine subfields of the Early Treatment Diabetic Retinopathy Study (ETDRS) grid (e.g., a central 1-mm circle), respectively, representing the foveal area, and inner and outer rings of diameters 3 and 6 mm, (Figures 1A,B). The inner and outer rings fell to four quadrants, i.e., superior, nasal, inferior, and temporal (Figures 1A,B). The foveal avascular zone (FAZ) area was automatically segmented and then quantified with the OCTA software. The quality of every image was manually checked by a professional ophthalmologist (XB), and the random eye was scanned according to time constraints. Only images of good quality were covered, and images with poor signal strength (signal strength index <7) or motion artifacts visible were excluded here. The observer was blinded to the clinical diagnosis of the participants.

### Statistics Analysis

For all analyses in this study, SPSS version 25 (SPSS Inc., Chicago, IL, United States) was employed. Sex differences between the groups were compared by performing the *Pearson* Chi-square test. The general features including age, systolic blood pressure (SBP), and ophthalmologic parameters (IOP, BCVA, AL) were expressed as mean ± the *SD*s. The OCTA measures were expressed as median (25% quartile, 75% quartile). Student’s *t*-test was performed to assess the difference between PD and HC groups. To compare the differences between PD subgroups, the results of this study were analyzed with one-way ANOVA based on the LSD *Post Hoc* test. The Benjamini–Hochberg Adjusted *p*-value was calculated to control the false discovery rate (FDR), and the FDR was set at 0.05. Variables not complying with a normal distribution were analyzed by performing a non-parametric test to compare the differences between groups. Correlations between data were tested by Spearman’s correlation. The differences between groups were considered to show statistical significance when *p* < 0.05.

## Results

A total of 182 participants were recruited in this study, which covered 67 HC, 50 H-Y I stage patients, 46 H-Y II stage patients, and 19 H-Y III stage patients. No statistical difference was identified in age distribution (*p* = 0.151), sex (*p* = 0.889), SBP (*p* = 0.645), intraocular pressure (IOP) (*p* = 0.583), best-corrected VA (BCVA) (*p* = 0.839), and axial lengths (AL) (*p* = 0.357) between PD and HC group (Table 1). Moreover, no significant differences were reported among the subgroups of patients with PD. Furthermore, the patients with PD with the H-Y III stage exhibited longer disease duration, higher UPDRS, UPDRS III, NMSS scores, and lower MMSE scores (*p* < 0.05). Table 1 lists the general demographics, ophthalmic features of all participants, and clinical parameters.

**TABLE 1:** Demographic characteristics of all enrolled participants.

Variables	All participants		*p*^a^* values*	PD			*p*^b^* values*
	PD	HC		H-Y I	H-Y II	H-Y III	
Eyes, N	115	67	NA	50	46	19	NA
Age, y	63.61 ± 6.92	62.01 ± 7.74	0.151*	62.08 ± 6.73	65.35 ± 7.07	63.47 ± 6.41	0.068^‡^
Sex, male, N (%)	51.00 (44.35%)	29.00 (43.28%)	0.889^†^	19.00 (38.00%)	24.00 (52.20%)	8.00 (42.10%)	0.369^†^
IOP, mmHg	15.53 ± 2.02	15.36 ± 2.07	0.583*	15.42 ± 2.19	15.74 ± 1.97	15.32 ± 1.67	0.655^‡^
SBP, mmHg	141.2 ± 19.31	142.6 ± 20.53	0.645*	140.7 ± 20.47	141.2 ± 18.98	142.7 ± 17.83	0.931^‡^
BCVA, logMAR	0.35 ± 0.13	0.35 ± 0.13	0.839*	0.33 ± 0.13	0.35 0.13	0.38 ± 0.13	0.478^‡^
AL, mm	21.97 ± 0.57	22.06 ± 0.60	0.357*	21.94 ± 0.57	22.01 ± 0.56	21.98 ± 0.63	0.826^‡^
Duration, y	6.00 (4.00–8.00)	NA	NA	4.00 (3.00–6.00)	7.00 (5.00–9.00)	9.00 (7.00–12.00)	**<0.001** **
UPDRS	33.00 (22.00–54.00)	NA	NA	20.50 (12.75–26.25)	44.50 (31.75–56.00)	69.00 (55.00–76.00)	**<0.001** **
UPDRS III	17.00 (11.00–31.75)	NA	NA	11.00 (6.75–15.00)	27.50 (17.75–37.25)	45.00 (34.00–54.00)	**<0.001** **
NMSS	20.50 (9.00–43.75)	NA	NA	14.00 (6.00–23.50)	35.50 (15.75–50.00)	41.00 (27.00–75.00)	**<0.001** **
MMSE	28.00 (26.00–29.00)	NA	NA	28.00 (27.00–30.00)	27.00 (25.75–29.00)	26.50 (23.50–29.00)	**0.012** **

Significant results appear in bold. p^a^: p-value for comparison between HC and PD; p^b^: p-value for comparison among the three PD subgroups. *Student’s t-test; ^†^Pearson’s χ^2^ test; ^‡^One-way ANOVA; **Non-parametric test.

*PD, Parkinson’s Disease; HC, healthy controls; IOP, intraocular pressure; SBP, systolic blood pressure; BCVA, best-corrected visual acuity; AL, axial lengths; UPDRS, Unified Parkinson’s Disease Rating Scale; UPDRS III, Unified Parkinson’s Disease Rating Scale III; NMSS, Non-motor Symptoms Scale; MMSE, Mini-Mental State Examination.*

### Macular Vessel Density Parameters

**FIGURE 2:**
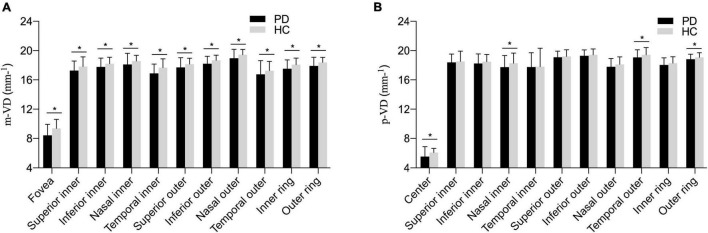
The comparison of vessel density parameters among different PD subgroups **(A)** in the macula area; **(B)** in the peripapillary area. Compared with HC groups, macular vessel density (m-VD) in the PD group was significantly declined in all quadrants. Additionally, peripapillary vessel density (p-VD) was found significantly decreased in the fovea, nasal inner (NI), temporal outer (TO), and outer ring regions in the PD group, *p < 0.05.

Figure 2A and Table 2 presents the m-VD parameters of all participants. The results demonstrated that compared with HC groups, m-VD in the PD group declined noticeably in all subfields (all *p* < 0.05) (Figure 2A and Table 2). Moreover, quadrantal analyses indicated the FAZ area decreasing significantly in the SCP of the patients with PD in comparison with the HC group [0.36 (0.32, 0.39) mm^2^ in healthy eyes vs. 0.29 (0.26, 0.33) mm^2^ in patients, *p* < 0.001] (Figures 1C,D). As revealed from the results of the one-way ANOVA analysis, the H-Y III stage group showed significantly decreased m-VD in the fovea, SI, II, NI, TI, TO, and lower FAZ area (all *p* < 0.05). However, a statistically significant difference was not identified in the SO, IO, and NO subfield for the comparison of PD subgroups (Table 2). In addition, results suggested that patients with PD with H-Y I stage showed FAZ area noticeably declining early (*p* < 0.05). Moreover, the m-VD in all grids in patients with PD with H-Y III were significantly lower than those in healthy eyes. Specific to the whole inner ring and outer ring index, statistically significant differences were reported for the PD and HC group, or PD subgroups (*p* < 0.05) (Table 2).

**TABLE 2:** Macular vessel density in HC and patients with PD.

Macular parameters	All participants		*p*^a^* values*	H-Y I	H-Y II	H-Y III	*p*^b^* values*	*p values*		
	HC	PD						H-Y I *vs.* HC	H-Y II *vs.* HC	H-Y III *vs.* HC
Fovea	9.25 (8.48, 10.50)	8.20 (7.70, 9.10)	**<0.001** *	8.50 (7.90, 9.53)	8.20 (7.70, 9.03)	7.90 (7.00, 8.70)	**0.035** ^†^	0.186*	**<0.001** ^‡^	**<0.001** *
SI	18.00 (17.28, 18.73)	17.40 (16.80, 18.10)	0.014*	17.85 (17.00, 18.45)^‡⁣‡^	17.35 (17.00, 18.00)^‡⁣‡^	16.50 (14.50, 17.30)**,^†⁣†^	**<0.001** ^‡^	0.591*	**0.012** ^‡^	**<0.001** *
II	18.40 (17.55, 18.93)	18.00 (17.40, 18.60)	**0.038^‡^**	18.20 (17.68, 18.73)^‡⁣‡^	18.00 (17.50, 18.60)^‡⁣‡^	16.60 (15.00, 17.70)**,^†⁣†^	**<0.001** ^‡^	0.575*	0.172*	**<0.001** ^‡^
NI	18.70 (18.00, 19.20)	18.40 (17.80, 18.80)	**0.026^‡^**	18.60 (18.18, 18.90)^‡⁣‡^	18.40 (18.00, 18.90)^‡⁣‡^	17.20 (16.00, 18.00)**,^†⁣†^	**<0.001** ^‡^	0.595^‡^	0.174*	**<0.001** ^‡^
TI	17.80 (16.98, 18.60)	16.90 (16.50, 17.70)	**<0.001** *	17.25 (16.70, 17.93)^‡⁣‡^	16.80 (16.48, 17.90)^‡⁣‡^	15.90 (14.90, 17.00)**,^†⁣†^	**<0.001** ^‡^	0.292*	**0.012** *	**<0.001** *
SO	18.25 (17.68, 18.70)	17.90 (17.20, 18.50)	**0.020^‡^**	18.10 (17.48, 18.50)	17.80 (17.20, 18.50)	17.10 (16.00, 18.40)	0.073^‡^	0.348^‡^	0.103*	**0.003** ^‡^
IO	18.70 (18.43, 19.00)	18.40 (17.80, 18.90)	**0.006^‡^**	18.55 (17.98, 18.93)	18.35 (17.80, 19.00)	17.80 (16.80, 18.80)	0.116^‡^	0.219^‡^	**0.026** *	**0.003** ^‡^
NO	19.60 (18.90, 19.83)	19.10 (18.40, 19.80)	**0.021^‡^**	19.10 (18.60, 19.80)	19.40 (18.38, 19.80)	18.70 (17.00, 19.50)	0.108^‡^	0.182*	0.069*	**0.002** ^‡^
TO	17.50 (16.70, 18.03)	17.10 (16.30, 17.80)	**0.046^‡^**	17.60 (16.98, 18.05)^†⁣†,^^‡⁣‡^	16.85 (16.28, 17.40)**	16.00 (14.30, 17.10)**	**<0.001** ^‡^	0.617*	**0.033** *	**<0.001** ^‡^
FAZ	0.36 (0.32, 0.39)	0.29 (0.26, 0.33)	**<0.001** *	0.31 (0.28, 0.34)^‡⁣‡^	0.28 (0.26, 0.32)^‡⁣‡^	0.24 (0.22, 0.27)**,^†⁣†^	**<0.001** ^†^	**<0.001** *	**<0.001** *	**<0.001** *
Inner ring	18.10 (17.58, 18.80)	17.70 (17.20, 18.30)	**0.005^‡^**	18.05 (17.60, 18.35)^‡⁣‡^	17.75 (17.28, 18.30)^‡⁣‡^	16.50 (15.40, 17.50)**,^†⁣†^	**<0.001** ^‡^	0.324*	**0.030** *	**<0.001** ^‡^
Outer ring	18.50 (18.10, 18.80)	18.20 (17.50, 18.70)	**0.020^‡^**	18.30 (17.78, 18.80)^‡⁣‡^	18.20 (17.60, 18.60)	17.20 (16.40, 18.80)**	**0.032** ^‡^	0.500^‡^	**0.012** *	**0.002** ^‡^

Significant results appear in bold. p^a^: p-value for comparison between HC and PD; p^b^: p-value for comparison among the three PD subgroups. *Student’s t-test; ^†^One-way ANOVA; ^‡^Non-parametric test.

Intergroup significant difference in Post Hoc Analysis: **Compared with H-Y I stage group; ^†⁣†^Compared with H-Y II stage group; ^‡⁣‡^Compared with H-Y III stage group. The Benjamini-Hochberg Adjusted p-value was calculated to defend the false discovery rate (FDR), and the FDR was set at 0.05.

*PD, Parkinson’s disease; HC, healthy controls; SI, Superior inner; II, Inferior inner; NI, Nasal inner; TI, Temporal inner; SO, Superior outer; IO, Inferior outer; NO, Nasal outer; TO, Temporal outer; FAZ, Foveal avascular zone.*

### Peripapillary Vessel Density Parameters

The p-VD parameters of all participants are presented in Figure 2B and Table 3. As indicated from the result, no significant difference of p-VD was found in all the subfields of the peripapillary scan between PD and HC groups (Figure 2B and Table 3). Meanwhile, no statistically significant difference was reported in p-VD among PD subgroups (*p* > 0.05) (Table 3).

**TABLE 3:** Peripapillary vessel density in HC and patients with PD.

Peripapillary parameters	All participants		*p*^a^* values*	H-Y I	H-Y II	H-Y III	*p*^b^* values*	*p values*		
	HC	PD						H-Y I *vs.* HC	H-Y II *vs.* HC	H-Y III *vs.* HC
Center	6.10 (4.80, 6.95)	5.10 (3.70, 6.40)	0.055^‡^	5.70 (4.60, 6.40)	4.50 (3.00, 7.10)	4.40 (2.50, 5.50)	0.682	0.396^‡^	0.154^‡^	0.110^‡^
SI	18.75 (17.68, 19.70)	18.60 (17.60, 19.20)	0.499*	18.60 (17.60, 19.10)	18.60 (17.50, 19.35)	18.40 (17.60, 18.70)	>0.999	0.695*	0.671*	0.238^‡^
II	18.60 (18.00, 19.03)	18.50 (17.80, 19.13)	0.470^‡^	18.20 (17.88, 18.73)	18.80 (17.40, 19.40)	18.30 (17.30, 18.70)	0.908	0.269*	0.809^‡^	0.382*
NI	18.50 (17.80, 19.30)	17.90 (17.10, 18.70)	0.081*	18.05 (17.08, 18.70)	18.20 (17.00, 19.15)	17.60 (17.20, 18.50)	>0.999	0.638*	0.330^‡^	0.275*
TI	18.10 (16.53, 19.85)	17.90 (16.98, 19.00)	0.561^‡^	17.90 (16.45, 19.10)	18.00 (17.05, 19.05)	17.70 (17.00, 18.90)	>0.999	0.953*	0.934*	0.440^‡^
SO	19.50 (18.70, 19.80)	19.30 (18.68, 19.60)	0.463*	19.25 (18.68, 19.53)	19.30 (18.50, 19.75)	19.30 (18.70, 19.50)	>0.999	0.692*	0.681*	0.419*
IO	19.60 (19.20, 19.90)	19.40 (18.90, 19.80)	0.401*	19.55 (18.98, 19.83)	19.30 (18.85, 19.90)	19.20 (18.90, 19.60)	0.955	0.634*	0.579*	0.419*
NO	18.10 (17.75, 18.90)	18.15 (17.20, 18.53)	0.114*	18.00 (17.08, 18.60)	18.20 (17.25, 18.50)	17.90 (17.50, 18.40)	0.964	0.301*	0.268*	0.327*
TO	19.60 (18.90, 20.20)	19.30 (18.50, 19.70)	0.074*	19.25 (18.45, 19.90)	19.40 (18.45, 19.65)	19.00 (18.50, 19.40)	>0.999	0.333*	0.201*	0.390*
Inner ring	18.30 (17.68, 19.13)	18.10 (17.40, 18.80)	0.158*	18.00 (17.40, 18.93)	18.30 (17.55, 18.90)	18.00 (17.70, 18.30)	>0.999	0.356*	0.740^‡^	0.251^‡^
Outer ring	19.20 (18.70, 19.50)	18.95 (18.40, 19.30)	0.116*	18.90 (18.40, 19.33)	19.00 (18.25, 19.30)	19.00 (18.50, 19.10)	>0.999	0.572*	0.275*	0.206*

*p^a^: p-value for comparison between HC and PD; p^b^: p-value for comparison among the three PD subgroups.*

*Student’s t-test; ^†^One-way ANOVA; ^‡^Non-parametric test.

*The Benjamini–Hochberg Adjusted p-value was calculated to defend the false discovery rate (FDR), and the FDR was set at 0.05. PD, Parkinson’s disease; HC, healthy controls; SI, Superior inner; II, Inferior inner; NI, Nasal inner; TI, Temporal inner; SO, Superior outer; IO, Inferior outer; NO, Nasal outer; TO, Temporal outer; FAZ, Foveal avascular zone.*

### Correlation Between Vessel Density and Other Parameters

**FIGURE 3:**
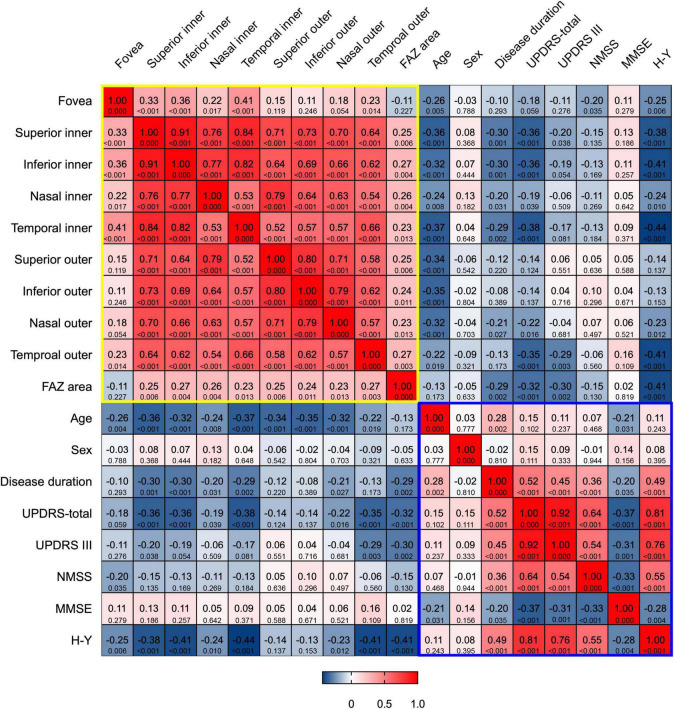
Heat map of correlation between m-VD and clinical parameters. There was a significant negative correlation between all subfields m-VD and age. In addition, there was a negative correlation between the m-VD of some subfields and disease duration, UPDRS-total, UPDRS III, NMSS scores, and H-Y stage. The **top row** of each cell is the Spearman correlation coefficients, and the **bottom row** is the corresponding *p*-value. The yellow and blue box represented the correlations among OCT parameters, and among clinical parameters, respectively.

The correlation of m-VD of patients with PD with clinical parameters was analyzed, (e.g., age, sex, disease duration, UPDRS, UPDRS III, NMSS, MMSE scores, as well as H-Y stage). Spearman’s correlation coefficients and corresponding *p*-value are presented in Figure 3. As indicated from the results, all subfields m-VD indicated a significant negative correlation with age. In addition, we observed that there was a negative correlation was identified between the m-VD of some subfields and disease duration, UPDRS total, UPDRS III, NMSS scores, and H-Y stage (*p* < 0.05). However, no statistically significant differences were noted for the correlation between the m-VD and sex and MMSE scores (both *p* > 0.05).

## Discussion

Retinal microvasculature variations in patients with PD have been rarely studied, and the relevant results are inconsistent. As reported by Shi et al. (2020), PD displayed a lower retinal capillary skeleton of SCP in all the quadrants as compared with HC in our study. However, Gulmez Sevim et al. (2019) found that the diameter of the retinal vessel showed no change in patients with PD. Our study is the initial study to explore the change of retinal vessel density in patients with PD divided into three stages based on the H-Y stage.

In this study, compared with the HC group, the m-VD in SCP in all subfields was suggested to significantly decrease in patients with PD. Moreover, as revealed from the in-depth analysis, the H-Y III stage group had lower m-VD in the fovea, SI, II, NI, TI, TO, and lower FAZ area, whereas no statistically significant difference was identified in p-VD for PD subgroups. The FAZ size significantly declined in patients with PD with H-Y I stage compared with HC. Thereby demonstrating that the abnormal change could be detected at the early stage of patients with PD. These suggested that the FAZ area may be a more sensitive indicator to detect PD at the early stage (H-Y I stage). Moreover, as suggested from the results here, mid-stage (H-Y II and H-Y III) patients with PD tended to show greater generalized microvascular variations. Furthermore, a significant negative correlation was identified between m-VD and both the age and H-Y stage score.

Vascular impairment may account for the pathogenesis of PD (Guan et al., 2013). As reported by earlier studies, several neurological disorders including PD are associated with ischemia or cerebrovascular lesions (Alavi et al., 1998; Kalaria, 2000). Since the retina is a “window” to the brain, they share an analogous pathophysiological mechanism. Cerebral vascular variations in PD might spread to the retina and lead to retinal angiopathy in the microvascular network. Guan et al. (2013) revealed the disruption of capillary network in patients with PD (e.g., shorter length, less number, and fewer branches). It was inferred that intracellular oxidative stress (Cuenca et al., 2014) might critically contribute to capillary variations. According to existing studies, decreased dopamine (Harnois and Di Paolo, 1990) levels and pathologic α-synuclein deposits (Zhang et al., 2015) were found in the wall of the blood vessels in the retina, especially in the ganglion cells located in SCP (Ortuño-Lizarán et al., 2018). As suggested from quantification studies of the inner retinal capillary in PD, microvascular variation in patients with PD displays an association with the neuronal loss in the inner retina (Miri et al., 2015; Kromer et al., 2016; Kwapong et al., 2018), which may comply with this study and explain why m-VD was found to decrease in SCP and retinal neurodegeneration.

The high oxygen consumption determines the vessel structure in the macular region (Yu et al., 2019), meanwhile, the energy metabolism in the retina is mainly on the neurons. In addition, the blood from the relatively high density and sophisticated capillary network around the foveola, supplying to the macula (Yu et al., 2019). It means neuron loss associated with the decrease of m-VD, and macula especially in the fovea VD might be a more sensitive region to the change in patients with PD.

The foveal avascular zone (FAZ) located in the fovea, surrounded by the continuous capillary network of the retina, had no capillary structure. Such regions contained DA neurons in the human retina. Berkowitz and Patel (2020) reported that patients with PD have smaller FAZ areas than healthy controls, complying with our findings. The mechanism underlying the FAZ area reduction is suggested to damage foveal DA neurons, which promotes vasculogenesis from the capillary surround (Miri et al., 2015). On the contrary, whether alterations of capillary morphology and pathology act in parallel on DA neurons and microglial cells surrounding the FAZ, was not confirmed. Same with most neurodegenerative diseases, vascular variation may contribute to the neuronal loss (Guan et al., 2013). This is consistent with our results that the deficiency of DA (Harnois and Di Paolo, 1990) and smaller FAZ size accompanied by the reduction in foveal vessel density indicated the damaged and incomplete parafoveal capillary arcade in the macula.

According to the results of this study, macular vascular variation might occur before the variation in p-VD. This might mainly be attributed to the fact that the macular SCP largely originates from the retinal circulation, complying with the results here. However, the peripapillary radial peripapillary capillary (RPC) receives perfusion, and the circulation from the choroidal ciliary vessels (Zhang et al., 2019). Moreover, the loss of the capillaries might not be detected by OCTA, as impacted by the overlap of the multilayered capillaries in RPC. Previous research has demonstrated, thinner nerve fibers concentrate on the temporal side of the optic nerve head (ONH), the thinner of the nerve fibers (Mikelberg et al., 1989), the shallower decline of the retinal nerve fiber layer (RNFL), this would be common to the vascular structure of blood circulation of these regions (Jo et al., 2019). This might be another reason. To date, this is the first study that analyzed peripapillary VD in patients with PD.

An inverse correlation between m-VD in the SCP and age, disease duration, UPDRS scores, NMSS scores, and H-Y stage was another novel finding in the report here. It is therefore predicted that the higher the age, UPDRS scores, NMSS scores, H-Y stage, and longer disease duration, the lower the density in the SCP. Existing studies reported no association between microvascular densities and H-Y scales (Kwapong et al., 2018; Shi et al., 2020). The possible explanation for this discrepancy with the measures here might result from our smaller study population, comprising the early and mid-stage of PD in this study. Meanwhile, age substantially has an impact on VD in the macula. The relationship between the m-VD and age reflected the vessel density decreased with age, as previous studies reported (Jo et al., 2019).

However, some limitations remained in our investigation. Due to poor patient cooperation and OCTA high inspection requirements for subjects, this study lacked advanced patients with PD, however, we can still observe a decreasing trend of m-VD in this study as the severity of PD increased. Meanwhile, this is consistent with the results of our correlation analysis that m-VD is inversely related to UPDRS scores. In addition, the use of anti-Parkinson’s disease medicine was not considered in patients with PD, and this assessment of disease severity would be biased. Moreover, this study was cross-sectional, and the m-VD changes could not be captured over time. Thus, further longitudinal, larger sample study (e.g., the advanced stage patients with PD were included) should be performed subsequently.

## Conclusion

The results here suggested that abnormal variations could be detected with OCTA at the early stage of patients with PD. In addition, as the disease progresses, m-VD showed a broader decrease, which is associated with age and disease severity. Furthermore, m-VD and FAZ might be a more sensitive index than p-VD to detect the variations. As revealed from the mentioned results, OCTA is a potential biomarker to detect the changes in different clinical stages of PD by visualizing retinal structure *in vivo* directly. OCTA can act as a valuable tool for early diagnosis and progression for PD.

## Data Availability Statement

The raw data supporting the conclusions of this article will be made available by the authors, without undue reservation.

## Ethics Statement

The studies involving human participants were reviewed and approved by the Research Ethics Committee of Xiangya Hospital, Central South University. The patients/participants provided their written informed consent to participate in this study.

## Author Contributions

BX and XW collected and analyzed the data and wrote the manuscript. BX, XW, JG, HX, and BT collected participants’ data and discussed the results. BX performed an OCT images scan of all participants. LS and BJ conceived the project, designed the research, supervised the data collection and analysis, and revised the manuscript. All authors contributed to the article and approved the submitted version.

## Conflict of Interest

The authors declare that the research was conducted in the absence of any commercial or financial relationships that could be construed as a potential conflict of interest.

## Publisher’s Note

All claims expressed in this article are solely those of the authors and do not necessarily represent those of their affiliated organizations, or those of the publisher, the editors and the reviewers. Any product that may be evaluated in this article, or claim that may be made by its manufacturer, is not guaranteed or endorsed by the publisher.
